# Tannin as Antidote to Strychnine

**Published:** 1860-08

**Authors:** 

**Affiliations:** Vienna


### TANNIN AS ANTIDOTE TO STRYCHNINE.

BY PROFESSOR KURZAC, OF VIENNA.

From want of a reliable antidote, the treatment in cases of poisoning by strychnine hitherto consisted principally in endeavoring to evacuate the poison, to combat the frightful spasmodic symptoms by narcotics, and to re-establish respiration, when it finally ceased, by artificial means. Donne proposed iodine, chlorine, and bromine, as antidotes to strychnine; Garrod, Rand, Morson, and Falck recommended prepared animal charcoal; but the efficacy of these substances has been neither tested sufficiently by experiment nor proved by experience. The same is true in regard to tannin, and the astringent vegetables containing it, their infusions, decoctions, etc. Although they recommended themselves by the fact that tannin forms chemical compounds, insoluble in water, with strychnine and other poisonous alkaloids, it seemed very probable that these products might be redissolved in the stomach and intestines, and thus be rendered capable of absorption; the virtue of tannin as antidote to strychnine was, therefore, considered very doubtful.

With a view to subject this matter to a thorough examination, and to ascertain the efficacy of tannin in preventing and allaying the symptoms of poisoning by strychnine, Professor Kurzac made a series of experiments on rabbits and dogs. At the end of his interesting and highly important memoir, he states that the results of his investigation permit him to draw the following conclusions:

1. Tannin, if administered in time, is an excellent chemical antidote to strychnine.

2. The doubt, whether the precipitate formed by tannin in a solution of strychnine, although insoluble in water, would not be redissolved by the gastric and intestinal juice, and the strychnine thus reobtain its poisonous properties, is solved by these experiments on rabbits and dogs in a complete and highly gratifying manner.

3. The successful results in dogs and rabbits justify the expectation that tannin would suspend the poisonous action of strychnine also in man, even in cases where the evacuation of the tannate of strychnine, formed in the stomach, could not be accomplished.

4. These experiments show that twenty to twenty-five times the quantity of tannin is required in order to suspend the poisonous action of the strychnine. In cases of poisoning it will be, however, advisable to administer a relatively larger proportion, as a part of the antidote will be absorbed by the usual contents of the stomach, particularly by gelatin.

5. As tannin has proved to be an antidote to nitrate of strychnia, which is much more soluble in water, there is so much greater reason to hope that it will be successful in poisoning by pure strychnia which dissolves in water with much difficulty.

6. The same successful result is to be expected from its administration in poisoning by the hard and tough nux vomica, which imparts the poison to aqueous fluids, but gradually and not very rapidly.

7. Tannin is a so much more valuable antidote in poisoning by strychnine, as galls in which it is contained can be readily procured, and thus be administered without much loss of time. They are easily reduced to a powder, which is given, mixed with water. Another advantage is obtained by the vomiting which it is liable to produce. In the mean time an infusion or decoction of powdered galls may be prepared.

On an average, Turkish galls contain fifty, and the Illyrian galls twenty per cent. of tannin. At least one drachm of the former and two drachms and a half of the latter, are therefore required to neutralize one grain of strychnine introduced into the stomach, but in general, especially if there is vomiting, a much larger quantity should be administered.

8. Another readily obtained substance containing tannin is Chinese tea, the efficacy of which in poisoning by strychnine, is confirmed by our experiments. But these experiments (7 and 8) have also shown that, in a decoction of tea leaves, we cannot count upon the whole amount of tannin contained in them. In poisoning by a larger dose, it would therefore be necessary to administer so large an amount of green tea that the antidote itself might produce poisonous effects. One decigramme ($\frac{1}{3}$ grain) of nitrate of strychnine requires, as our experiments prove, ten drachms (600 grains, 40 teaspoonfuls) of green tea, which, according to Poligot's analysis, contain about fifteen grains of caffein. Tea is therefore applicable only in poisoning by smaller doses, but may otherwise be useful as adjuvant.

9. The efficacy of roasted coffee as chemical antidote to strychnine seemed to be much inferior. The amount of caffeo-tannic acid contained in coffee is, according to Payen, 3.5 to 5.0 per cent. But our experiments (9, 10, and 11) show that the decoction evidently contains a much smaller quantity of undecomposed tannic acid than this per centage would justify us in assuming. The decoction of 180 grains of roasted Cuba coffee (being adequate to 200 grains of the raw coffee, which should contain at least six grains of tannic acid) produced, according to the ninth experiment, merely a delay and diminution of the poisonous effect of 0.13 grain of nitrate of strychnine. In the tenth and eleventh experiments, 300 grains of raw coffee, which weighed, after roasting, 267 and 264 grains, and should have contained at least nine grains of tannic acid, had furnished a decoction which, as antidote to 0.13 grain of strychnine, was nearly inert, only delaying the appearance of the symptoms for a little while.

10. From unroasted coffee, so inconsiderable an amount of tannin is extracted, by boiling, that the employment of its decoction for our purpose is out of question.

11. Oak bark (of Quercus robur and Q. pedunculata) contains, according to Gerber, 8.5 per cent. of tannic acid, and imparts it readily to aqueous fluids. It deserves attention in poisoning by strychnine so much the more, as it can be procured without much delay, especially in the country. What has been said about the administration of galls equally applies to the use of the powder and decoction of this bark.

12. On account of their frequent occurrence and the large amount of tannin they contain, we have to mention in this connection: acorns (from Quercus robur and Q. pedunculata) with 9 per cent; the bark of the horse-chestnut, with 8 per cent.; willow bark, with 5½ per cent.; and the green hull of walnuts. The radix tormentillæ, with 17 per cent.; rad. caryophyllatæ, with 31 per cent.; and rad. bistortæ, are still richer in tannin, but can rarely be procured without much loss of time.

13. The solubility of the precipitate, produced by tannin in a solution of strychnine, by acetic, citric, and tartaric acid, (vide experiments with the same,) show the necessity of avoiding vegetable acids during the treatment of poisoning by strychnine with tannic acid.

14. The same applies to the internal use of alcohol and alcoholic remedies.

15. The reported experiments on rabbits have sufficiently proved that more active voluntary movements excite the spasms usually produced by strychnia, even when they otherwise would not have made their appearance. In treating cases of poisoning by strychnine, it is therefore highly important *to prohibit, as much as possible, all voluntary movements, and to avoid violent excitement of any other kind.—Zeitschrift der K. K. Gesellschaft der Aerzte zu Wien, March* 12, 1860.

